# Primary Ewing Sarcoma of the Thyroid—Eight Cases in a Decade: A Case Report and Literature Review

**DOI:** 10.3389/fendo.2017.00257

**Published:** 2017-10-27

**Authors:** Paweł Kabata, Sonia Kaniuka-Jakubowska, Wanda Kabata, Joanna Lakomy, Wojciech Biernat, Krzysztof Sworczak, Janusz Jaśkiewicz, Maciej Świerblewski

**Affiliations:** ^1^Department of Surgical Oncology, Medical University of Gdańsk, Gdańsk, Poland; ^2^Department of Endocrinology and Internal Medicine, Medical University of Gdańsk, Gdańsk, Poland; ^3^Wanda Kabata General Practice, WJS Ltd., Gdańsk, Poland; ^4^Department of Pathomorphology, Medical University of Gdańsk, Gdańsk, Poland

**Keywords:** thyroid sarcoma, Ewing sarcoma, extraosseus ewing sarcoma, ewing’s sarcoma of the thyroid, thyroid ewing sarcoma

## Abstract

Sarcomas represent less than 1% of all malignant tumors found in the thyroid. Of these, primary extraosseoussarcoma has been reported only a few times in the past decade. We present the case of a 34-year-old male who had a fast-growing hard mass in the lower left neck. FNA was inconclusive. Core needle biopsy revealed the diagnosis of an Ewing sarcoma/primitive neuroectodermal tumor. Mutation of EWSR1 was confirmed using the FISH method. Following treatment by neoadjuvant chemotherapy, we observed clinical, radiological, and finally histopathological remission. This was followed by a left-sided isthmolobectomy with unilateral cervical lymph node dissection by lateral lymphadenectomy, which revealed no residual disease. Posttreatment radiotherapy was administered but discontinued upon the patient’s request. After 18 months of observation, the patient had no recurrence or metastasis and required l-thyroxine supplementation. We discuss our case using a comparative literature review to the few other known case reports.

## Introduction

Sarcomas represent only a few percent of all neoplasms found in the thyroid, appearing in less than 1% of all patients treated for malignant thyroid tumors (1). The most commonly found histological types are angiosarcoma, malignant hemangioendothelioma, malignant fibrous histiocytoma, leiomyosarcoma, and fibrosarcoma (2).

Ewing sarcoma is most often found in children, being the second most common malignant bone tumor in this age group. However, its extraskeletal localization is found in approximately 20% of patients, most often in the thorax and abdomen (43%), with only 8% of these tumors being located in the head and neck region (3).

We present the case of a patient who underwent successful multimodal treatment for extraosseous Ewing sarcoma located in the thyroid. Additionally, a comparative literature review of other reported cases was performed and is discussed.

## Materials and Methods

For the literature review, PubMed, Google Scholar, and Medline databases were searched using the terms: *thyroid sarcoma, Ewing sarcoma, extraosseous Ewing sarcoma*. The search covered publications from 2007. Only papers describing primary tumors were considered and four full-text articles were identified. Two other cases were extracted from a case series describing Ewing family tumors of the head and neck region although, thorough information regarding these cases is limited (4). Detailed available information regarding the reviewed cases is presented in Table 1.

**Table 1:** Clinicopathological features of reviewed cases.

Reference	Adapa et al. (5)	Chan et al. (6)	Chan et al. (6)	Maldi et al. (7)	Chirila et al. (8)	Bishop et al. (4)	Bishop et al. (4)	Present case
Age	9	23	67	66	48	19	36	34
Sex	F	M	F	M	M	M	F	M
Tumor presentation	Smooth, non-tender, rubbery mass	Asymptomatic right neck mass	Increase of a previously observed nodule	Single nodule discovered in a follow-up PET scan after treatment of B-cell lymphoma	Acute obstructive respiratory failure secondary to thyroid swelling	Growing neck mass	Goiter	Fast-growing hard mass
Lymph nodes	(−)	(−)	(−)	(−)	N/A	N/A	N/A	(−)
Ultrasound	Solid, heterogenous, hypervascular mass. Left lobe and isthmus normal	47 mm right thyroid mass	Increase from 4 to 40 mm	Solid 45 mm heterogenous, hypervascular	N/A	N/A	N/A	Pathological mass filling the whole left lobe 79 mm × 44 mm × 84 mm
Computed-tomography/MRI	Heterogenous mass with peripheral enhancement. Displacement of trachea, compression of IJV	N/A	N/A	N/A	N/A	N/A	N/A	Pathological mass 58 mm × 60 mm. Deviation of trachea. Non-specific lymph nodes in neck and upper mediastinum
Dissemination	(−)	(−)	(−)	N/A	Metastases to brain after 1 month	(−)	(−)	(−)
Thyroid function tests	Normal	Normal	N/A	Normal	N/A	N/A	N/A	Normal
Fine needle biopsy	Suggestion of hematologic malignancy with nodal involvement	Not performed	Follicular neoplasm or lesion suspicious for follicular neoplasm	Thyroid localization of lymphoma	Giant B-cell non-Hodgkin lymphoma	N/A	N/A	Small-cell malignant neoplasm, most likely of hematologic origin
Core needle biopsy/postoperative pathology	N/A	Round epithelioid cells arranged in nests; tumor cells mixed with normal parenchyma or forming nests with no thyroid parenchyma; round to elongated nuclei with stippled chromatin; extensive angiolymphatic invasion	N/A	Malignant tumor of possible neuroectodermal origin	Extraosseous Ewing sarcoma/primitive neuroectodermal tumor	Uniform small cells; areas of nested growth with prominent fibrosis separating tumor lobules; colonization of underlying follicles; zones of microcystic growth set in a prominent myxoid stroma	Uniform small cells; areas of nested growth with prominent fibrosis separating tumor lobules; colonization of underlying follicles	Small-blue-round-cell tumor suggesting Ewing sarcoma/primitive neuroectodermal tumor
IHC	Vimentin (+) NSE (+) CD99/O13 (+) hematopoietic markers (−)	CD99 (+) synaptophysin (+) chromogranin (+) TTF (−) PAX8 (−) CK7 (−) CK20 (−) CK56 (−) CEA (−) desmin (−)	CD99 (+) vimentin (+) pankeratin (+) AE1/3 (+) TTF (−) PAX8 (−) CEA (−) calcitonin (−) CD56 (−) CK7 (−) CK20 (−) CK5/6 (−) chromogranin (−) synaptophysin (−)	Vimentin (+) CD99 (+) NSE (+) synaptophysin (+)	N/A	CD 99 (+) CK (+) synaptophysin (−) chromogranin (−) S100 (focally+) actin (focally+) desmin (−) NUT-1 (−)	CD 99 (+) CK (+) synaptophysin (+) chromogranin (focally+) S100 (−) actin (−) desmin (−)	Synaptophysin (+) CD99 (+) PAS (+) CKAE1/AE3 (+) Cam 5.2 (+) TTF1 (−) calcitonin (−) Bcl2 (−) LCA (−)
ESWR1 translocation	(+)	(+)	(+)	(+)	N/A	(+)	(+)	(+)
Surgery	Right isthmolobectomy with resection of strap muscles	Thyroidectomy followed by lateral and central neck dissection	Right lobectomy followed by complete left thyroidectomy	Total thyroidectomy	Nearly complete resection of the tumor with laryngectomy and resection of five tracheal rings	Performed, not otherwise specified	Performed, not otherwise specified	Left isthmolobectomy with cervical lymphadenectomy
Chemotherapy	Vincristine, doxorubicin, cyclophosphamide, iphosphamide, etoposide, and mesna	Vincristine, actinomycin D, cyclophosphamide, doxorubicin, ifosfamide, etoposide	Cyclophosphamide, doxorubicin, vincristine, ifosfamide, mesna, etoposide	None	CHOP primary lymphoma diagnosis, etoposide, and carboplatin	N/A	N/A	Doxorubicin, cyclophosphamide, vincristine, iphosphamide, etoposide, and mesna
Pre/postsurgery	Presurgery	Postsurgery	Postsurgery	Disqualified because of significant comorbidities	Postsurgery	Postsurgery	Postsurgery	Presurgery
Response to treatment	Good	N/A	Good	Metastases in follow-up	Died from brain metastases after one cycle	Awaiting chemotherapy	Awaiting chemotherapy	Complete clinical response
Follow up	6 years	N/A	22 months	8 months	1 month	0 month	0 month	18 months
Radiotherapy	Yes	Yes	No	No	No	N/A	N/A	Not completed

*(+), positive result*.

*(−), negative result*.

*N/A, information not available*.

Of the reviewed cases (*N* = 7), most were adult males (5/7; 57.1%), 9–67 years, and all displayed growing neck masses without coexisting lymphadenopathy. All patients underwent surgical treatment. Standard chemotherapy regimen for Ewing sarcoma was administered to three of the seven patients, both pre- and postoperatively. One patient did not receive systematic treatment because of significant comorbidities. Information regarding chemotherapy for two of the patients was not available.

Diagnosis of Ewing sarcoma/primitive neuroectodermal tumor was made based on either a core needle biopsy or surgical specimen, and the reports confirmed that six out of seven patients displayed EWSR1 mutation on fluorescence *in situ* hybridization. One case lacked information on its EWSR1 mutation status. The initial cytological reports uncovered suspected hematologic malignancy in four of the five patients who had fine needle biopsy performed.

Our patient signed an informed consent and granted permission for use of his medical history in this publication.

## Case Presentation

**Figure 1:**
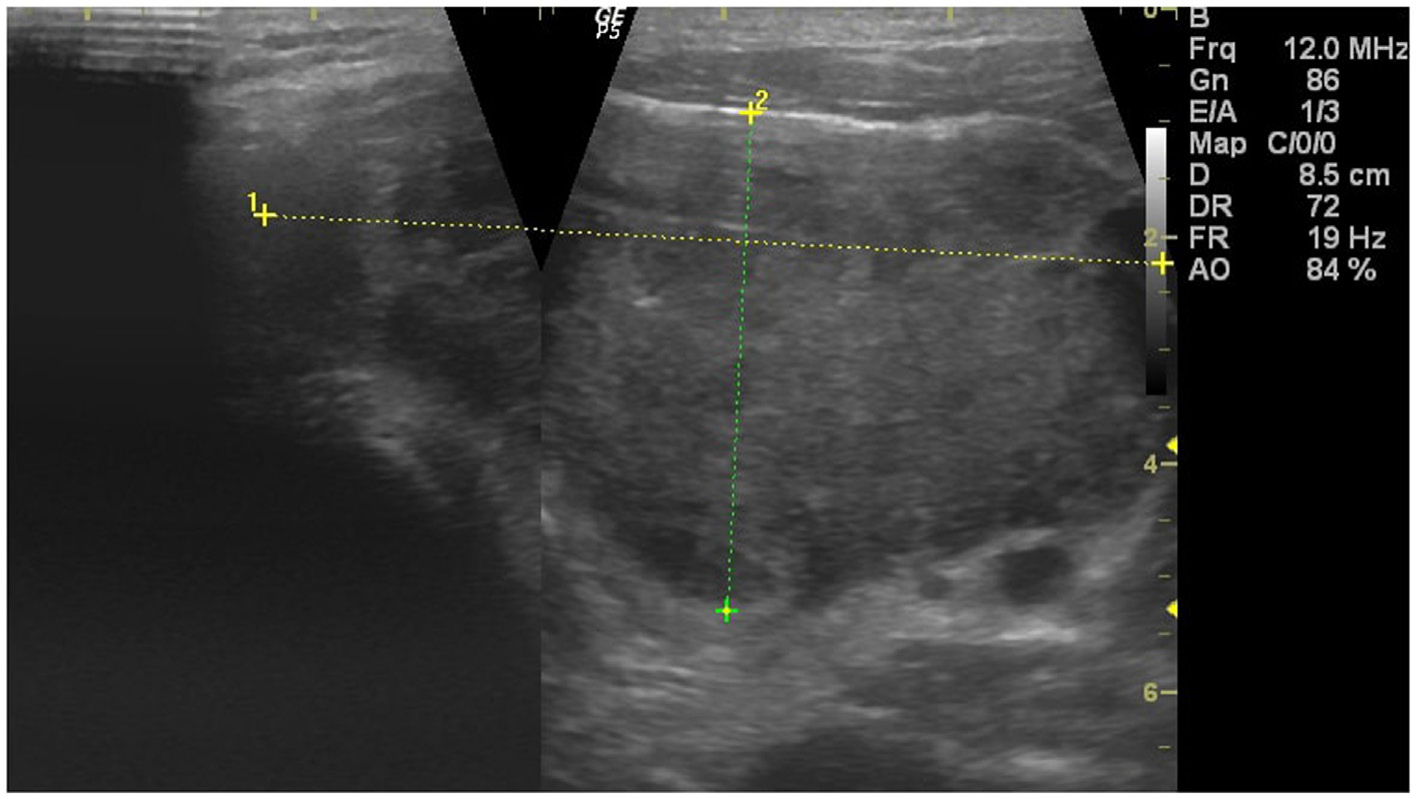
Ultrasound appearance of the tumor.

Our patient, a 34-year-old male, working as a chef, visited a general practitioner with signs of upper respiratory tract infection, sore throat, and difficulties in swallowing. After undergoing a typical prescribed treatment for a cold, the patient’s symptoms reportedly resolved. After 3 weeks and no signs of the disease, the symptoms recurred, however, this time, a palpable, fast-growing hard mass in the lower left neck was observed. An ultrasound of the neck revealed a large, solid, hypoechogenic, hypervascular mass with a heterogenous echostructure, and irregular margins in the left lobe of the thyroid. The tumor measured 79 mm × 44 mm × 84 mm and filled the entire left lobe, but was limited to the thyroid without signs of infiltration of the capsule or surrounding tissues (Figure 1). No pathological lymph nodes were found, except for a few slightly enlarged oval-shaped ones lacking in pathological blood supply. Static elastography assessment showed normal stiffness of the tissue.

**Figure 2:**
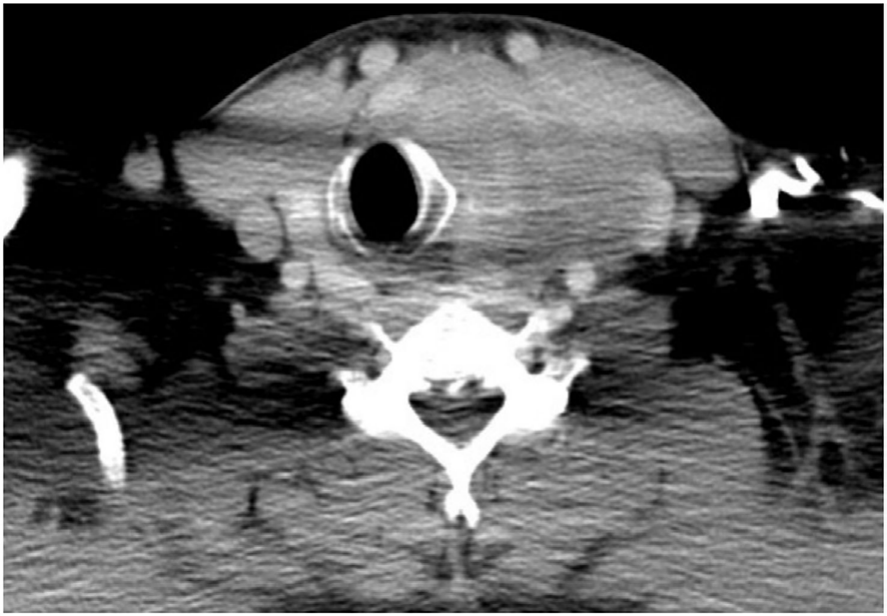
Computed-tomography appearance of the tumor.

**Figure 3:**
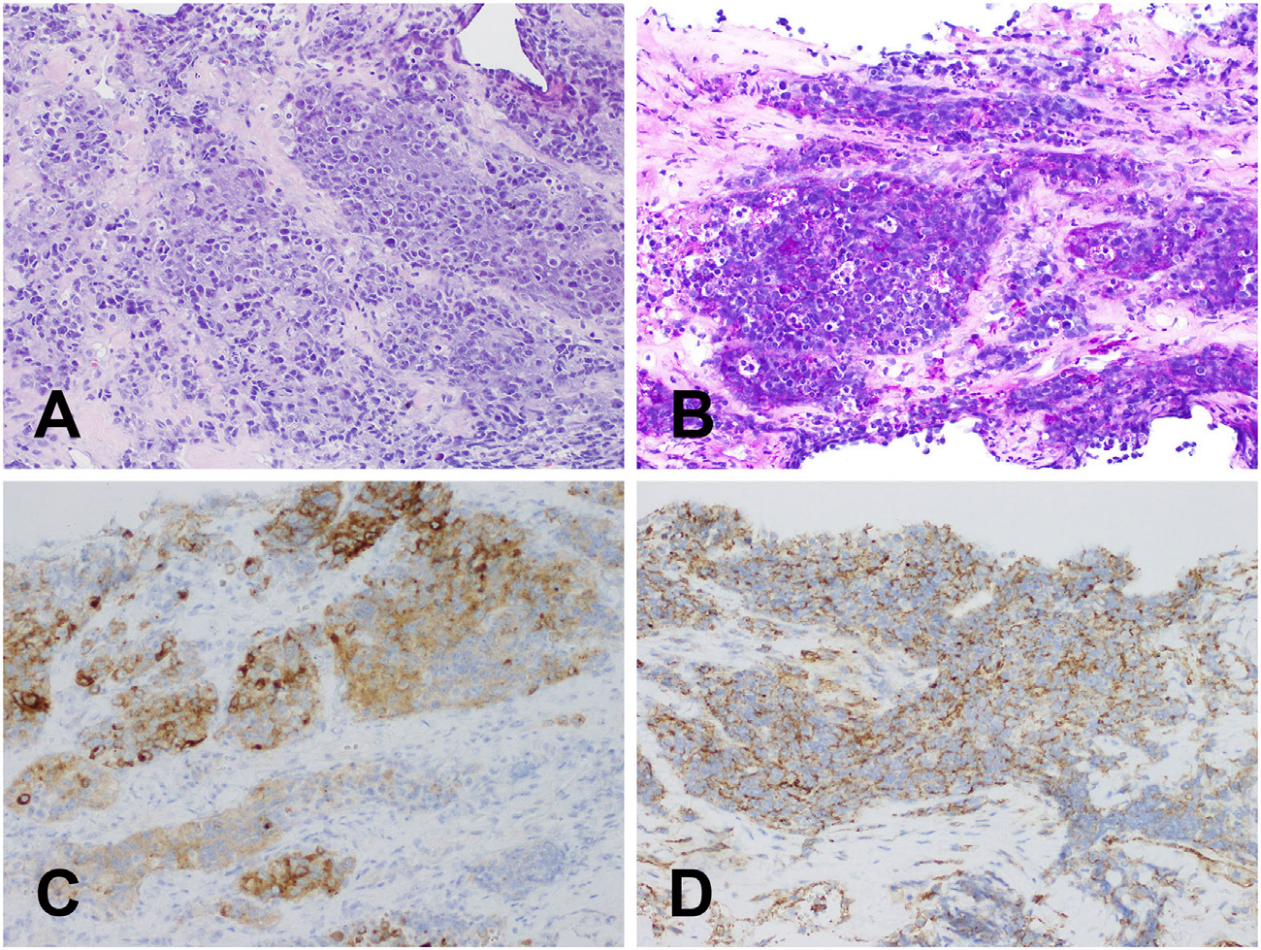
Histologic examination. Core needle biopsy of the tumor: **(A)** magnification ×200; hematoxylin–eosin staining—small-blue-round-cell tumor; **(B)** magnification ×200; PAS—positive staining; **(C)** magnification ×200; S100—positive staining; **(D)** magnification ×200; CD99—positive staining.

The patient was referred to the Department of Surgical Oncology, Medical University of Gdansk for further evaluation. A contrast-enhanced computed-tomography (CT) of the neck, thorax, and abdomen revealed a mass in the left lobe of the thyroid 58 mm × 58 mm × 60 mm, causing tracheal deviation (Figure 2). A number of non-specific, oval-shaped lymph nodes up to 14 mm were found in the neck and upper mediastinum, with no other pathological lesions being found. A fine needle biopsy was then performed, which revealed a small-celled malignant neoplasm, most likely of hematological origin. Laboratory markers—TSH, T3, T4, LDH were in reference range, respectively: TSH 0.64 μU/ml (0.34–0.94), fT3 4.9 pmol/l (2.63–5.7), fT4 14.28 pmol/l (9.01–19.05), LDH 201 U/l (125–220) with CEA and calcitonin being negative. As the patient had no previous history of neoplastic disease, core needle biopsy of the tumor was performed. It revealed a small-blue-round-cell tumor with immunophenotype: synaptophysin (+), CD99 (+), PAS (+), CKAE1/AE3 (+), Cam 5.2 (+), TTF1 (−), calcitonin (−), Bcl2 (−), and LCA (−), which suggested a Ewing sarcoma/primitive neuroectodermal tumor (Figure 3). Mutation in EWSR1 was confirmed using the FISH (fluorescence *in situ* hybridization) method.

**Figure 4:**
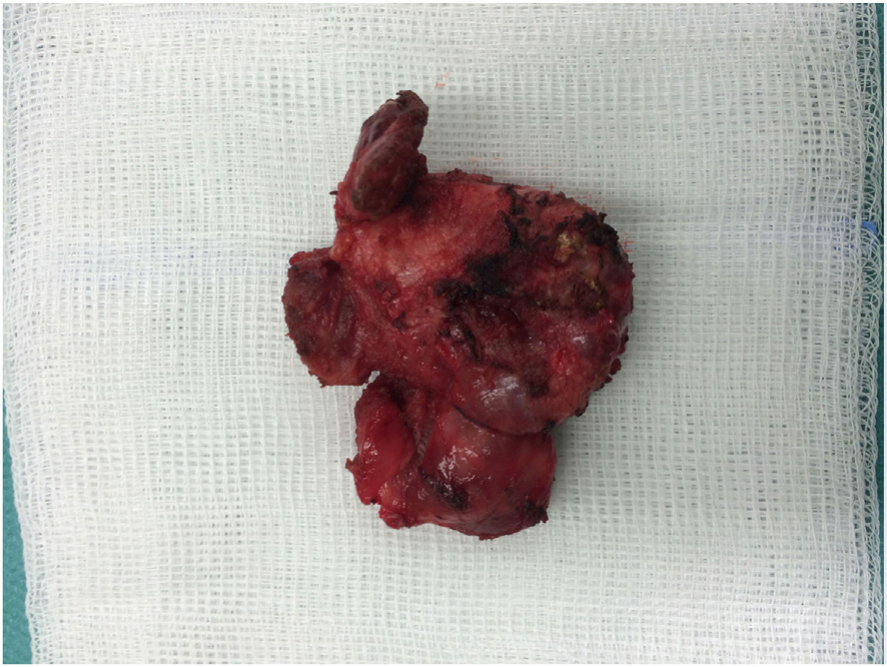
Postoperative specimen—left lobe of the thyroid with the isthmus.

After a multidisciplinary team consultation, which included surgical, medical, and radiation oncology opinions, the patient qualified for neoadjuvant chemotherapy with doxorubicin 25 mg/m^2^, cyclophosphamide 1,200 mg/m^2^, vincristine 2 mg, iphosphamide 3.6 g, etoposide 200 mg, and mesna. After six cycles of chemotherapy, a complete, clinical response was observed and the patient was referred for surgery. Left-sided isthmolobectomy with unilateral cervical lymph node dissection of groups II, III, IV, and V was performed, confirming clinical response to treatment (Figure 4). Postoperative pathological examination reported complete response to therapy with no pathological lesions found in the thyroid. All 28 lymph nodes removed were free from malignant cells.

During the postoperative period, mild left-sided Horner’s syndrome was observed, which resolved spontaneously after 2 weeks. The patient was discharged home on the fifth day following surgery and was then followed up during his visits to the surgical outpatient clinic until wound healing was complete. He then started adjuvant radiotherapy, which was discontinued because of dysphagia. The patient has currently undergone oncological follow-up and observation for 18 months with no signs of recurrence or dissemination of the disease. 16 months postsurgically, when TSH level was of 6.6 μU/ml (0.34–4.94) we started supplementation of l-thyroxine.

## Discussion

Ewing sarcoma/primitive neuroectodermal tumors are a group of small-, round-celled tumors, with different degrees of neuroectodermal differentiation (7). These neoplasms are most commonly found localized in the skeletal system of pediatric patients. Primary extraosseous Ewing sarcomas of the thyroid are extremely rare and have only been reported a few times in the past decade. Other types of sarcoma such as angiosarcoma or malignant hemangioendothelioma are found more frequently, but still play a minor role in thyroid oncology.

Diagnosis of thyroid Ewing sarcoma based on its clinical and radiological features or even fine needle biopsy seems to be challenging. The clinical manifestation of malignancy can be a rapidly growing mass, sometimes described as swelling on the neck (5, 6, 9). The disease displays no features of thyroid gland dysfunction, therefore, the thyroid hormones are within reference ranges. Using an ultrasound, a solid, heterogeneous, hypervascular soft tissue mass within the thyroid was found, which creates a difficulty of differentiating it from other thyroid malignancies (5, 7, 9). Performing a fine needle biopsy did not obtain a proper diagnosis in any of the reported cases (Table 1). As shown in our review, using a cytological assessment, these tumors might mimic hematological malignancies, therefore, a core needle specimen should be taken to obtain a proper diagnosis and start adequate treatment.

Because of the scarcity of such cases, no guidelines regarding optimal treatment options are available. All the reviewed cases where detailed information of treatment was available underwent the typical treatment regimen for Ewing sarcoma; however, the timing of chemotherapy differed. According to NCCN guidelines for treatment of Ewing sarcoma, neoadjuvant multi-agent chemotherapy should be administered prior to local therapy (surgery or radiotherapy) (10). As expected, in the treatment of Ewing sarcoma of other organs, our case confirms the efficacy of neoadjuvant therapy. Despite the initially large tumor mass (CT 58 mm × 58 mm × 60 mm), following isthmolobectomy with unilateral cervical lymph node dissection, we did not find any malignant cells in the histopathological thyroid specimens. Previous case reports refer only to postsurgery chemotherapy, with the sole exception of neoadjuvant therapy, but the case lacks a response assessment from before surgery. With such a limited number of cases, it is impossible to assess which approach is better; however, we at least know that both are effective and neoadjuvant therapy can provide total remission. Regardless of the timing, chemotherapy is crucial for treatment of this neoplasm.

Standard surgical treatment of Ewing sarcoma does not cover lymphatic resection (10). From the cases reviewed, cervical lymphadenectomy was performed for only one patient, only because medullary thyroid cancer was initially suspected. Postoperatively, lymph node metastases were found. The diagnosis was then verified as being Ewing sarcoma. A complimentary lymphatic resection was performed, which revealed metastases and the patient was referred for adjuvant chemotherapy. Only in our case was information regarding the lymph node ratio after neoadjuvant treatment available, and it showed no metastases in the cervical lymph nodes. In the other cases, where only wide surgical resection was performed, no nodal recurrences were observed. On the other hand, Chinese surgeons reported nodal recurrence of synovial sarcoma of the thyroid in a patient where neck dissection was omitted (9). Therefore, based on the reviewed material, we cannot tell whether lymphadenectomy could be omitted for treatment of Ewing sarcoma of the thyroid. With limited data, coming mostly from case reports and case series, it is impossible to state clear recommendations.

## Conclusion

Extraosseous Ewing sarcoma of the thyroid is an extremely rare tumor. Neoadjuvant therapy can provide total histopathological remission before surgery. Because very few literature cases are described, it is difficult to establish a set standard of care. Our case seems to be a confirmation that the standard suggested treatment regimens for Ewing sarcoma can be safely applied for such patients.

## Author Contributions

PK, SK-J, MS, and WK managed the case. PK and SK-J drafted the manuscript. PK, SK-J, MS, KS, and JJ reviewed the manuscript. JL and WB prepared histopathological results.

## Conflict of Interest Statement

The authors declare that the research was conducted in the absence of any commercial or financial relationships that could be construed as a potential conflict of interest.
